# Patterns of Cone-Beam Computed Tomography (CBCT) Utilization by Various Dental Specialties: A 4-Year Retrospective Analysis from a Dental and Maxillofacial Specialty Center

**DOI:** 10.3390/healthcare9081042

**Published:** 2021-08-13

**Authors:** Silvina Friedlander-Barenboim, Wafi Hamed, Avraham Zini, Noam Yarom, Itzhak Abramovitz, Harry Chweidan, Tamar Finkelstein, Galit Almoznino

**Affiliations:** 1 The Sheba Medical Center, Tel-Hashomer, Oral Medicine Unit, Ramat Gan 5265601, Israel; silfriedbar@gmail.com (S.F.-B.); Noam.Yarom@sheba.health.gov.il (N.Y.); tamar@vas.co.il (T.F.); 2 Oral and Maxillofacial Center, Department of Prosthodontics, Israel Defense Forces Medical Corps, Tel-Hashomer, Ramat Gan 02149, Israel; wafihamed@gmail.com (W.H.); harryc100@gmail.com (H.C.); 3 Hadassah Medical Center, Department of Community Dentistry, Faculty of Dental Medicine, Hebrew University of Jerusalem, Jerusalem 91120, Israel; AviZ@hadassah.org.il; 4 The Maurice and Gabriela Goldschleger School of Dental Medicine, Tel-Aviv University, Tel Aviv 6139001, Israel; 5 Hadassah Medical Center, Department of Endodontics, Faculty of Dental Medicine, Hebrew University of Jerusalem, Jerusalem 91120, Israel; Itzhakab@hadassah.org.il; 6 Department of Orthodontics, The Maurice and Gabriela Goldschleger School of Dental Medicine, Tel-Aviv University, Tel-Aviv 6139001, Israel; 7 Hadassah Medical Center, Department of Oral Medicine, Sedation & Maxillofacial Imaging, Faculty of Dental Medicine, Hebrew University of Jerusalem, Jerusalem 91120, Israel; 8 Hadassah Medical Center, Big Biomedical Data Research Laboratory, Faculty of Dental Medicine, Hebrew University of Jerusalem, Jerusalem 91120, Israel

**Keywords:** cone-beam computed tomography (CBCT), maxillofacial radiology, dental radiology

## Abstract

The study aimed to analyze the uses of cone-beam computed tomography (CBCT) in the diagnosis and treatment in various dental specialties. This 4-year cross-sectional study analyzed the records of 1409 individuals who underwent a CBCT at the Oral and Maxillofacial Center at Sheba Medical Center, Israel. The average age of the patients was 27.9 ± 11.5 (range: 9–86 years). Patients were referred for CBCT by the following departments: Oral and Maxillofacial Surgery (1063; 75.5%), Endodontics (182; 12.9%), Periodontology (122; 8.6%) and Orthodontics (42; 3.0%). Most CBCT radiographs evaluated the maxilla (774; 55.0%), followed by the mandible (481; 34.1%) and both (154; 10.9%). The target anatomical structures included: bone (694; 49.3%), teeth (307; 21.7%), and both jaws (408; 29.0%). The main indications for CBCT use were: assessment of anatomical structures and implant sites (787; 55.9%), determine root canals morphology (182; 12.9%), visualization of impacted teeth, tooth alignment, and localization (177; 12.6%), suspected cysts or tumors (148; 10.5%), evaluation of Temporomandibular joint disorders (106; 7.5%) and other reasons (9; 0.6%). In 279 (19.8%) of cases, the diagnosis changed following CBCT, mainly in Orthodontics tooth analysis (28 (66.7%); *p* < 0.001). Practitioners and health authorities should be aware of this baseline information regarding CBCT use in the diagnosis and assessment of various oral and maxillofacial pathologies, anomalies and tooth position relative to anatomic structures. Continuing research and publications of CBCT utilization and guidelines are recommended.

## 1. Introduction

Along with clinical examination, radiological imaging is essential for a complete diagnosis in dental medicine. Analysis of radiographic images is often essential in confirming or negating clinical findings or diagnoses. In dentistry, two-dimensional panoramic and periapical radiographic images are widely used. These radiographs contain essential information regarding the teeth, jaws, and more anatomical structures such as the maxillary sinuses, temporomandibular joints (TMJ), and even the nasal cavity [1,2].

Nevertheless, panoramic and periapical radiographs have some limitations, such as being limited to two dimensional imaging, distortion, and blurring [3]. In some cases, the broad coverage of the panoramic and periapical radiographs will therefore still be insufficient to obtain an accurate diagnosis or enable the dentist to perform a treatment plan [4].

According to most prevailing guidelines, three-dimensional cone-beam computed tomography (CBCT) imaging is recommended for patients for whom the diagnosis would otherwise remain uncertain or the treatment plan unclear [5,6,7]. Development of specialized CBCT scanners for use in dentistry started in the second half of the 1990s [8,9]. In 1997, Arai and colleagues [9] created a prototype-limited CBCT device for dental use that was dubbed Ortho-CT [9]. Two years afterwards, the device was successfully used to evaluate conditions such as impacted teeth, apical lesions, and mandibular and maxillary diseases, both before and after surgery [10]. In 2000, this technology was transferred to Morita Co., Ltd. Thereafter, Mozzo and colleagues [8] developed the Tom QR 9000, which was designed specifically to image the maxillofacial region. Soon after, the use of CBCT for dental, maxillofacial and ear–nose–throat applications started prospering, and, at present, CBCT is a widely used tool for several dental applications, such as implant planning, endodontics, maxillofacial surgery and orthodontics. The advantages of CBCT are its capabilities of volumetric jaw bone imaging, detecting exact morphology of roots and canals, and real relationship between adjacent anatomical structures at reasonable costs and relatively low doses, with comparative advantages of being compact, affordable, and therefore accessible with nearby or in-house equipment installation [11]. In some cases, as in implant therapy, CBCT has a role beyond diagnosis and is used for treatment planning, i.e., printing three-D models for surgical guide manufacturing [12]. However, there are some limitations to CBCT, such as the inability to accurately determine bone density [13,14]. The referral for CBCT imaging should always be guided by the pursuit of improved diagnostic accuracy and the prospect of an enhanced treatment plan. Preferably, the indications for a CBCT scan should be based entirely on case-related factors [7]. Yet, dentist-related factors might influence the request for CBCT as well. To address these issues, our primary objective was to analyze the patterns of CBCT utilization in a central Oral and Maxillofacial Dental Specialists Center. The specific objectives of this study were to analyze the utilization of CBCT in terms of indications for use, target anatomical structures, and impact on the diagnosis and delivery of treatment among various dental specialties.

## 2. Materials and Methods

The study included medical records of individuals who attended the Oral and Maxillofacial Specialists Center, Sheba Medical Center, Israel, between 1 January 2012 and 31 December 2015, and performed CBCT imaging using Carestream dental CS 9000, which is a combined machine including panoramic, cephalometric and 3D imaging, that consists of a single FOV (field of view) of 4×5 cm, which is a small FOV. The Sheba Medical Center is the largest medical center in Israel and one of the larger medical centers in the Middle East. The Oral and Maxillofacial Center is a specialist center that manages the treatment of dental and oral pathologies referred by dentists and physicians from clinics throughout the country. The center manages the treatment of both civilian and military populations in Israel. The Oral and Maxillofacial center includes a central Oral & Maxillofacial Radiology unit as well as specialized departments that provide state-recognized residency programs in Oral and Maxillofacial Surgery, Orthodontics, Endodontics, and Periodontology.

### 2.1. Ethical Approval

The study adheres to the STROBE guidelines. All procedures performed in studies involving human participants were following the ethical standards of the Sheba Medical Center (SMC) Institutional Review Board (IRB), (approval number: 0237-13-SMC) and with the 1964 Helsinki declaration and its later amendments or comparable ethical standards. Since this study was a retrospective study that involved only medical records analysis, it was approved by the IRB with an exemption from obtaining informed consent.

### 2.2. Inclusion and Exclusion Criteria

Inclusion criteria were:

1. Patients who underwent CBCT at the Oral and Maxillofacial Center at Sheba medical center during the study period.

2. Available CBCT decoding.

3. Available clinical data and radiographic examination before and after the CBCT.4. Available CBCT decoding with a lack of relevant data was an exclusion criterion.

### 2.3. Data Collection

For each individual, several parameters were recorded:

1. Demographics: age (years), sex (men/women).
2. Referring department: Oral and Maxillofacial Surgery/Orthodontics/Endodontics/Periodontology.
3. The indication for referring the patient for CBCT as recorded in the medical file.
4. Target jaw evaluated: Maxilla/Mandible/both jaws.
5. Anatomical structures evaluated: teeth/bone/teeth and bone.
6. Did panoramic X-ray precede the CBCT? yes/no.
7. Did periapical X-ray precede the CBCT? yes/no.
8. The indications for patient referral for the CBCT: for diagnosis/for treatment planning.
9. Final diagnosis: as recorded in the medical file.
10. Did the interpretation of the CBCT change the diagnosis and/or treatment plan which was given prior to referral? yes/no.

### 2.4. Statistical Analysis

Analysis of the data was performed with the SPSS software version 25.0 (Chicago, Illinois, United States) and statistical significance was defined as a *p*-value < 0.05. Continuous variables were presented as means and standard deviations. The categorical variables were presented as percentages and frequencies.

Tests to assess associations between the referring department parameter as a dependent variable and the independent variables included: likelihood ratio for categorical variables.

## 3. Results

**Figure 1:**
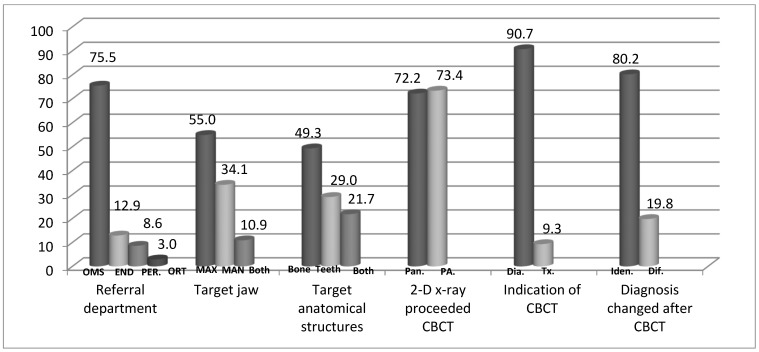
Distribution of The Study Population According to The Referring Department and Clinical Data Regarding The Patterns of CBCT Usage. OMS-Department of Oral and Maxillofacial Surgery, ENDO-Department of Endodontics, PER-Department of Periodontology, ORT-Department of Orthodontics, MAX-Maxilla, MAN-Mandible, Pan-Panoramic, PA-Periapical, DIA-Diagnostic, Tx-Treatment, IDEN-Identical, Dif-Different.

The study included 1409 medical records of patients who underwent CBCT during the study period. Of those, 1063 (75.4%) patients were men and 346 (24.6%) were women. The mean age of the patients was 27.9 ± 11.5 years and the age range was 9–86 years. Figure 1 presents the distribution of the study population according to the referring departments and according to the clinical data regarding CBCT usage.

**Referring department.** The main referring department was the Oral and Maxillofacial Surgery (OMS) Department (1063 patients, 75.5%), followed by the Department of Endodontics (182, 12.9%), the Department of Periodontology (122, 8.6%) and the Department of Orthodontics (42, 3.0%) (Figure 1).

**Target jaw.** When considering the whole study population including the four referring departments, the target jaw of the CBCT was the maxilla in the majority of the cases (774, 55.0%), followed by the mandible (481, 34.1%) and both the maxilla and the mandible (154, 10.9%) (Figure 1).

**Target anatomical structures**. The target anatomical structures of the CBCT included: bone (694, 49.3%), teeth (307, 21.7%) and both teeth and bone (408, 29.0%) (Figure 1).

**Imaging preceding the CBCT.** Periapical X-ray preceded the CBCT imaging in 1034 patients (73.4%), while panoramic X-ray proceeded the CBCT imaging in 1018 patients (72.2%) (Figure 1).

**The indication of the CBCT.** The CBCT was used as a part of the diagnostic process in 1278 patients (90.7%), while it was used as part of the treatment process or in the post-treatment evaluation in 131 patients (9.3%) (Figure 1).

**The input of the CBCT and its effect on the diagnosis and/or treatment plan.** The diagnosis and/or treatment plan did not change following the CBCT and matched the differential diagnosis and/or treatment plan that was given before the referral in 1130 patients (80.2%), while it was changed in 279 patients (19.8%), leading to change in the final diagnosis and/or treatment plan (Figure 1).

**Figure 2:**
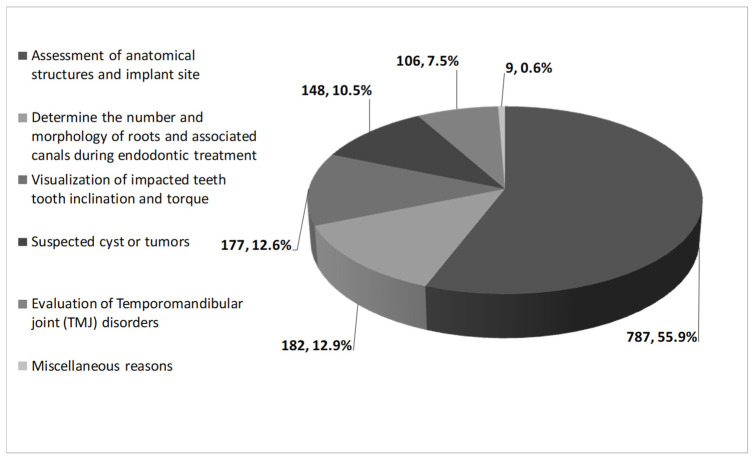
Indications for CBCT Use of The Study Population.

**The main indications for CBCT according to the differential diagnosis. **Figure 2 presents the main indications for CBCT use, which included: assessment of anatomical structures, height, and width of planned implant site (787 patients, 55.9%), determination of the number and morphology of roots and associated canals during endodontic treatment (182 patients, 12.9%), visualization of impacted tooth inclination and torque (177 patients,12.6%), suspected cyst or tumors (148 patients, 10.5%), evaluation of temporomandibular joint (TMJ) disorders (106 patients, 7.5%) and other miscellaneous reasons (9 patients, 0.6%) (Figure 2).

**Data on CBCT usage according to the referring department. **Following the description of data of the whole study population, we further analyzed these data stratified according to the referring department. Table 1 presents the data on CBCT usage according to the referring department.

**Target jaw according to the referring department.** The distribution of the target jaw that was scanned was statistically significantly different between the four departments (*p* < 0.001). All the four departments referred more cases for maxilla imaging, followed by mandible and both jaws, except for the Department of Periodontology. The Department of Periodontology referred to both jaws in 25.4% of the cases, while the Department of Orthodontics did not refer to imaging for both jaws in any of the cases (Table 1).

**Target anatomical structures according to the referring department.** The distribution of the anatomical structures that were scanned was statistically significantly different between the four departments (*p* < 0.001). All patients in the Departments of Orthodontics and Endodontics were referred for scans of teeth, while all patients in the Department of Periodontology were referred for scanning of the bone. The OMF department was the only department that referred for both teeth and bone CBCT scan (Table 1).

**Imaging preceding the CBCT according to the referring department. **The distribution of the imaging preceding the CBCT was statistically significantly different between the four departments. All patients in the Periodontology and the Endodontics departments were referred for a periapical X-ray before the CBCT, followed by the Department of Orthodontics (97.6%), compared to only 66.6% in the OMF department (*p* < 0.001). A panoramic radiograph was performed in most cases which were referred from the Orthodontics (95.2%) and the OMS (83.8%) departments, while the performance of panoramic radiograph before the CBCT was less frequent in the Periodontology (39.3%) and the Endodontics (21.4%) departments (*p* < 0.001) (Table 1).

**The indication of CBCT according to the referring department.** CBCT was used as a diagnostic tool in all patients in the Periodontology and the Endodontics departments, and in the vast majority of the cases in the Orthodontics (92.9%) and OMF (88.0%) departments (*p* < 0.001) (Table 1).

**The input of the CBCT and its effect on the diagnosis.** The percentage of cases that the diagnosis and/or treatment plan did not change following the CBCT and matched the differential diagnosis and/or treatment plan that was given before the referral in most cases was: OMS (82%), Endodontics (80.8%), and Periodontic (79.5%) departments, while in the Orthodontics department, the CBCT had more significant input on the diagnosis/treatment plan (66.7%), leading to a change in most cases (*p* < 0.001)(Table 1).

Final diagnoses of the study population according to department referring to CBCT.

**Figure 3:**
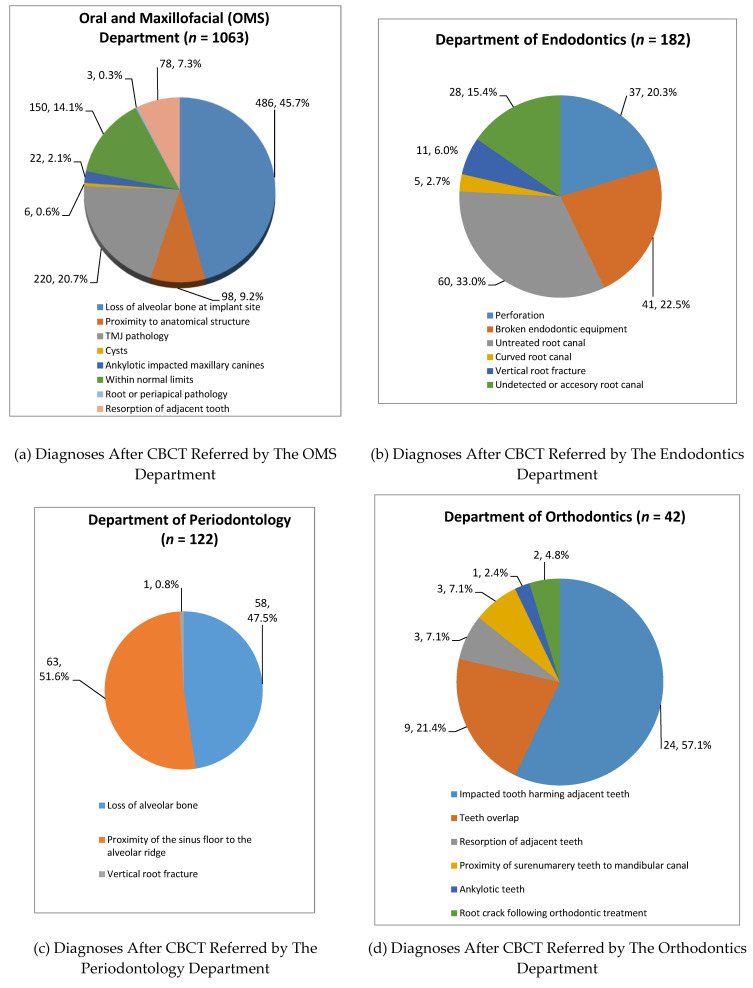
Final Diagnoses According to The Referring Department.

The final diagnoses according to the referring department are presented in Figure 3.

**Oral and Maxillofacial Department (*n* = 1063 patients) **(Figure 3, Panel A). The final diagnoses at the Department of Oral and Maxillofacial were:

(1) *Implant site evaluation* diagnosed as loss of alveolar bone at the implant site (486 patients, 45.7%)

(2) *Proximity to anatomical structures at implant sites* (98 patients, 9.2%) included: maxillary sinus floor proximity (49, 4.6%) and proximity to the mandibular canal (49, 4.6%).

(3)*Temporomandibular joint (TMJ) pathology*. 220 patients (20.7%) were diagnosed with TMJ pathologies, and these were comprised of: inflammatory hyperplasia (137, 12.9%), condylar hyperplasia (72, 6.8%) followed by osteoarthritis (6, 0.6%) and synovial disc derangement (5, 0.4%).

(4) *Cysts* (6, 0.6%) included: keratocyst 3 (0.3%), residual cyst 1 (0.1%), and radicular cysts in 2 (0.2%) cases.

(5)* Ankylotic impacted maxillary canines* were diagnosed in 22 (2.1%) of the cases.

(6) *Resorption of adjacent tooth *was diagnosed in 78 (7.3%) of the cases.

(7) Root or periapical pathology was diagnosed in 3 (0.3%) of the cases. This included 2 vertical root fracture cases and 1 condensing osteitis case.

(8) *Diagnosed as within normal limits (WNL)* included 150 cases (14.1%) of those 138 (13.0%) were diagnosed as ventilated maxillary sinuses without pathology, and no pathological findings were recorded in 12 (1.1%) of the cases.

**Department of Endodontics (*n* = 182) **(Figure 3, Panel B). The final diagnoses at the Department of Endodontics were: untreated root canal (60, 33.0%), broken endodontic instrument (41, 22.5%), perforation (37, 20.3%), undetected root canal (28, 15.4%), vertical root fracture (11, 6.0%), and curved root canals (5, 2.7%).

**Department of Periodontology (*n* = 122)** (Figure 3, Panel C). The final diagnoses at the Department of Periodontology were: loss of alveolar bone (58, 47.5%), the proximity of the sinus floor to the alveolar ridge (63, 51.6%), both diagnoses were part of implant site evaluation. There was one case (1, 0.8%) of a vertical root fracture.

**Department of Orthodontics (*n* = 42)** (Figure 3, Panel D). At the Department of Orthodontics, the following diagnoses were noted: impacted tooth harming adjacent teeth. (24 cases, 57.1%), referral to assess tooth overlap (9, 21.4%), the proximity of supernumerary teeth to the mandibular canal (3, 7.1%), resorption of adjacent teeth (3, 7.1%), root crack following orthodontic treatment (2, 4.8%), and ankylotic teeth (1, 2.4%).

## 4. Discussion

There are few research papers published in the English literature describing the analysis of the utility of CBCT in diagnosis, assessment, planning, and delivery of treatment. Our research was done to improve our understanding of the contribution and the limitations of CBCT imaging modality in the diagnosis and treatment planning by different oral and maxillofacial specialties.

### 4.1. The Impact of CBCT on The Diagnostic Process

There is wide agreement through many research publications that CBCT imaging had an added value in the diagnostic and determining treatment plan process [15,16]. Radic et al. [17] found that although the majority of the diagnoses were done correctly by the OMS and the orthodontic residents, patients were also referred for CBCT imaging for more information.

There is currently no definitive evidence to support the standard use of CBCT for postoperative evaluation [18,19,20]. For example, Jacobs summarized, in a systematic review, that there is currently no definitive evidence to support the standard use of CBCT for postoperative evaluation of peri-implant bone, and recommended that at this time, with certain exceptions, intraoral radiography should remain the main diagnostic imaging modality in monitoring implants post-operatively [20]. Moreover, even regarding intraosseous lesions, when screening for recurrence after complete enucleation or resection, panoramic radiographs is thought to be a sufficient process [18,21]. The same applies to the use of CBCT in the evaluation of orthognathic surgery outcome, where only few studies exist to support the use of CBCT over the long-term follow-up period [18]. Considering the lack of benefit, and the risk associated with exposure to radiation, the ALARA principle, “as low as reasonably achievable”, states that even if a small dose is used, if receiving that dose has no direct benefit, the clinician should try to avoid it.

In particular, during the COVID-19 pandemic, extraoral panoramic and CBCT were suggested to be prioritized over intraoral radiography due to less involvement with intraoral secretions and less paperwork. However, their disadvantages such as lower resolution, artifacts from movement and metallic restorations, as well as a much higher radiation dose CBCT, makes them no alternative to intraoral radiography [22,23]. It is therefore recommended to use intraoral radiography during the pandemic and to ensure proper disinfection of the patient’s protective collar and apron and before developing the X-rays to disinfect them with suitable disinfectant distributed with disposable paper according to the CDC guidelines [24].

### 4.2. Targeted Jaw and Anatomical Structure in CBCT

In the present study, the maxilla was scanned in most of the cases (55.0%), in line with previous reports by other researchers who reported a similar prevalence of approximately 50% of cases [25]. Others have also reported that CBCT was used mainly for implant site evaluation, in particular, in the posterior maxilla in cases that may require a maxillary sinus floor lift [26,27]. In our study, all four departments referred cases for CBCT of the maxilla more than the mandible. This trend could be attributed to the fact that there are many anatomical structures in the mid-face making CBCT necessary in the process of diagnosis and treatment planning. The Department of Periodontology referred more cases for CBCT of both jaws compared to the other departments, to assist assessing the loss of alveolar bone that is common in both jaws in periodontal cases [28,29]. The Department of Orthodontics referred cases for the assessment of one jaw to determine localized complexity or diagnosis.

The Endodontic and the Orthodontic departments referred cases for CBCT imaging where the target structure was tooth-related because of the pattern of cases dealing with tooth pathology or complexities beyond that of bone-related or other anatomical structures. However, the OMS Department deals with pathologies of the whole maxillofacial complex, and therefore referred cases for CBCT to scan teeth, bone, or both. The Department of Periodontology referred cases to determine the volume of the alveolar bone as an implant pre-surgical planning or periodontal disease evaluation. The use of 2D imaging before the CBCT differed between departments. The department of OMS referred most cases for CBCT after initial panoramic imaging.

### 4.3. Final Diagnoses: Oral and Maxillofacial Surgery Department

The majority of CBCT scans were referred by the OMS department, as shown in previous research [30]. It can be explained by the fact that the goals of the different referral departments are not the same. Oral and maxillofacial surgeons and periodontists need to determine surgical planning approaches, such as in dental implantology. The CBCT scan is widely used in the assessment of implant sites. According to the consensus guidelines (European Academy of Dental and Maxillofacial Radiology (EADMFR-2009) [31], European Association for Osseointegration (EAO-2011) [31] and the American Academy of Oral and Maxillofacial Radiology (AAOMR-2012) [32]), there are clear indications for the use of CBCT in implant dentistry [33]. The practitioner should optimize the CBCT scan including the exposure protocols, the desired field of view (FOV), and the technologies to minimize radiation dosage to the patient [4]. Dagassan-Berndt et al. [34] found that, on average, greater implant length was more often planned based on panoramic X-ray, while wider implants were generally more frequently planned based on CBCTs. In the same research, it was reported that implant treatment planning based on panoramic and on CBCT images was equal in 50% to 67% of the cases. Compared with the actual surgery, CBCT revealed a higher agreement (46% for implant length), while for panoramic, 34.4% equal implant length was observed [34]. The overall advantage of using CBCT in implant dentistry is related to its ability to acquire detailed volumetric image data of the maxillofacial region for diagnostic and pre-surgical planning purposes [3,34].

### 4.4. Department of Endodontics

Currently, intraoral radiography is the imaging technique of choice for endodontic diagnosis and treatment. CBCT imaging is used mainly for the diagnosis of anatomic variants and pathologies such as variations in root canal anatomy, vertical root fracture, untreated canals, endodontic treatment complications, such as overextended root canal obturation material, separated endodontic instruments, localization of perforations, and root resorption [35,36,37]. In the present study, the most prevalent indication for CBCT use was the evaluation of untreated canals, usually because the canals are undetected in intra-oral radiographs. In line with our findings, Lo Giudice et al. [35] showed that the untreated root canal is also the main indication for referring for CBCT, which may be explained by their finding that they were not diagnosed with the two-dimensional X-rays in 30.6% of the cases. Moreover, a radiolucent area was detected in the CBCT exam in 46%, while the intraoral periapical X-ray exam was positive only in 18% [35].

In the present study, the diagnosis changed for endodontic referrals in 19.2% of the cases. However, previous studies have shown that CBCT may have a dramatic effect on the endodontic treatment plan and management compared to two-dimensional X-rays [38,39,40]. According to Ee et al. [38], the treatment plan changed following CBCT in 62% of the endodontic cases. Mota de Almeida et al. [39] and Rodriguez et al. [40] reported that, in 53% of the cases (28 out of 53 cases), the therapy plan changed after the CBCT examination, especially in cases referred to differentiate pathology from normal anatomy. Davies et al. [41] concluded that CBCT had an impact on the future management of retreatment cases when compared to two-dimensional X-rays. Indeed, in recent years since the data were collected, there has been a shift to lower radiation protocols and better diagnostic value in high-resolution protocol scans. Considering this, some researchers even suggested that CBCT may become the first choice for endodontic treatment planning and outcome assessment, especially when new scanners with lower radiation doses will be available [35,42]. The evolvement in the field, and the need to set rules and regulations for CBCT use in endodontics, led to the publication of guidelines for endodontic use of CBCT in 2015 by the AAE [36,43] and in 2019 by the ESE [37] for improving the rationale for referral to CBCT in endodontic use.

### 4.5. Department of Periodontology

In the present study, the Department of Periodontology used CBCT in the majority of the cases to assess alveolar bone loss and the proximity of the sinus floor to the alveolar ridge. Assessment of the loss of alveolar bone at the department of Periodontology was to determine the volume of the alveolar bone as an implant pre-surgical planning or periodontal disease evaluation. According to the literature, in periodontology, CBCT may display two-dimensional and three-dimensional images that are necessary for the diagnosis and treatment planning of intra-bony defects, furcation involvements, and buccal/lingual bone destruction [44,45]. Moreover, it was reported that CBCT and conventional periapical radiographs differed in measuring the height of the alveolar bone crest [46]. Indeed, a recent systematic review provided additional evidence for the accuracy of CBCT in assessing periodontal bone loss [29]. The need to set rules and regulations for CBCT use in periodontology led the American Academy of Periodontology to publish a consensus statement on selected oral applications for CBCT [26].

### 4.6. Department of Orthodontics

To the best of our knowledge, there is no information in the English literature on the percentage of CBCT use in the maxilla and the mandible separately in Orthodontics departments. In the present study, most of the cases in the Orthodontic department were referred to check the relations between the impacted tooth and adjacent structures, and the diagnosis was changed in the majority of the cases (66.7%) that were referred for CBCT imaging. Others also reported that the original treatment plans derived from 2-dimensional radiographs were changed for more than 25% of the impacted teeth when orthodontists viewed these teeth in CBCT images as opposed to the 2-dimensional radiographs typically used for this purpose [47]. Thus, the scientific evidence for the utility of CBCT both in refining diagnosis and modifying treatment plans for significant numbers of impacted teeth validates its use for most impacted teeth [48,49].

Currently, the use of CBCT for Orthodontic purposes applies to impacted teeth, cleft lip/palate, and orthognathic or craniofacial surgery patients. Other possible indications for the use of CBCT in orthodontic cases include supernumerary teeth, identification of root resorption caused by unerupted teeth, evaluating boundary conditions, TMJ degeneration, airway assessment, and progressive bite changes [50,51], albeit additional research is needed to establish adequate guidelines for the use of CBCT in the field of orthodontics.

### 4.7. Strength and Limitations of This Study

This study was conducted in a secondary/tertiary public health medical center, the largest center in Israel and one of the larger ones in the Middle East. We eliminated recall information bias by including records-based demographics and medical data, which do not rely on the patients’ reports nor on questionnaires delivered to doctors. This study collected data from four different dental disciplines and assessed many different parameters related to patterns of CBCT utilization by the different specialties. To date, there are not many publications in the literature that cover such a variety of data. In particular in Israel, to the best of our knowledge, this is the first study to assess utilization patterns by the different specialties. This kind of research may serve in the future to determine and strengthen guidelines for CBCT applications. Indeed, Schulze et al. recommended the publication of further evidence-based guidelines will help the clinician and radiologist on the best use of CBCT in clinical practice [52].

Although the study assessed the experience of the largest medical center in Israel concerning CBCT use by different dental specialties, this study represents the experience of one center, thus the study population is a convenience sample, which may limit the generalizability of the results. Moreover, while this study assessed a period of 4 years, there was an evolution in recent years towards high-resolution protocol scans with lower radiation protocol, which warrants continuous assessments of the changes in the patterns of CBCT usage by different specialties. Currently, CBCT machines are developing fast, as well as the software used for reconstructing the images; thus, better image quality is available in different protocols for use in most of the new CBCT machines in the market. For example, it is possible to select the size of the FOV as well as the imaging protocol, meaning high- or low-resolution protocols. Implementation of iterative reconstruction techniques can substantially reduce noise [53]. Novel motion correction algorithms [54] enable to reduce blur and artifacts caused by patient movement or by metal and hyperdense materials present in the ROI (region of interest). Future multicenter studies are warranted to assess the patterns and trends over time of CBCT use by dental practitioners among wider populations of patients.

## 5. Conclusions

The study analyzed the utility of CBCT in the diagnosis, assessment, planning, and delivery of treatment in various specialties in dentistry in an Oral and Maxillofacial center. According to our results, the vast majority of referrals for CBCT scans were from the OMS department. The most common indication was evaluating bone and teeth for implant planning, and most CBCT radiographs evaluated the maxilla. In almost 20% of the cases, the primary diagnosis and/or treatment plan was changed following the CBCT assessment, emphasizing the important role of CBCT in the diagnosis and treatment by different dental specialties.

To conclude, and in accordance with consensus guidelines of the international associations, practitioners must be aware of using CBCT, especially nowadays where the radiation dose is still significantly higher than 2-dimensional radiographs. In the future, when the radiation dose of CBCT lowers to similar values of 2-D imaging, it may be the imaging technique of choice in dentistry. Continuing research and publications of CBCT utilization and guidelines are recommended. Continuing research and publications of CBCT utilization and guidelines are recommended.

## Figures and Tables

**Table 1:** Clinical Data Regarding the Patterns of CBCT Usage According to Referring Department.

Variable		Referring department				*p* Value
		Oral and Maxillofacial Surgery (*n* = 1063)	Endodontics (*n* = 182)	Periodontology (*n* = 122)	Orthodontics (*n* = 42)	
Target jaw	Maxilla	577 (54.3)	101 (55.5)	73 (59.8)	23(54.8)	<0.001 ^
	Mandible	371 (34.9)	73 (40.1)	18 (14.8)	19 (45.2)	
	Maxilla and mandible	115 (10.8)	8 (4.4)	31 (25.4)	0 (0)	
Target anatomical structures	Teeth	83 (7.8)	182 (100)	0 (0)	42 (100)	<0.001 ^
	Bone	572 (53.8)	0 (0)	122 (100)	0 (0)	
	Bone and teeth	408 (38.4)	0 (0)	0 (0)	0 (0)	
Panoramic X ray proceeded the CBCT	Yes	891 (83.8)	39 (21.4)	48 (39.3)	40 (95.2)	<0.001 ^
	No	172 (16.2)	143 (78.6)	74 (60.7)	2 (4.8)	
Periapical X ray preceded the CBCT	Yes	689 (66.6)	182 (100)	122 (100)	41 (97.6)	<0.001 ^
	No	374 (35.2)	0 (0)	0 (0)	1 (2.4)	
Indication of CBCT	Diagnostic need	935 (88.0)	182 (100)	122 (100)	39 (92.9)	<0.001 ^
	Treatment need	128 (12.0)	0 (0)	0 (0)	3 (7.1)	
Diagnosis has been changed after CBCT	No	872 (82.0)	147 (80.8)	97 (79.5)	14 (33.3)	<0.001 ^
	Yes	191 (18.0)	35 (19.2)	25 (20.5)	28 (66.7)	

^ Likelihood ratio.

## Data Availability

Data sharing not applicable.

## Abbreviations

CBCT: Cone-beam computed tomography; OMS: Oral and Maxillofacial surgery; TMJD: Temporomandibular joint disorders.
